# Benthic Crustacean Digestion Can Modulate the Environmental Fate of Microplastics in the Deep Sea

**DOI:** 10.1021/acs.est.9b07705

**Published:** 2020-03-19

**Authors:** Alessandro Cau, Carlo Giacomo Avio, Claudia Dessì, Davide Moccia, Antonio Pusceddu, Francesco Regoli, Rita Cannas, Maria Cristina Follesa

**Affiliations:** † Dipartimento di Scienze della Vita e dell’Ambiente, Universitá degli Studi di Cagliari, Via Tommaso Fiorelli 1, 09126 Cagliari, Italy; ‡ Consorzio Interuniversitario per le Scienze del Mare, CoNISMa, ULR Cagliari, Cagliari 09126, Italy; § Dipartimento di Scienze della Vita e dell’Ambiente, Universitá Politecnica delle Marche, Via Brecce Bianche, 60131, Ancona, Italy; ∥ Consorzio Interuniversitario per le Scienze del Mare, CoNISMa, ULR Ancona, Ancona 60131, Italy

## Abstract

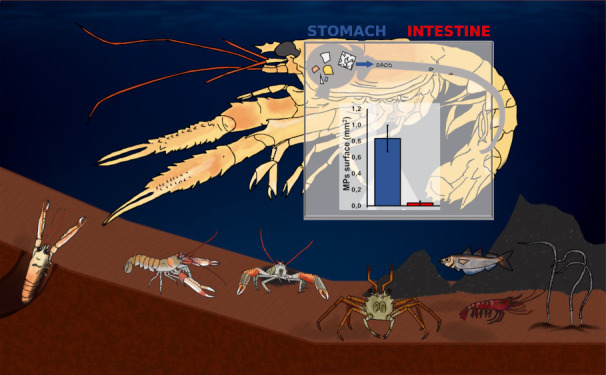

Microplastics (MPs) are ubiquitous contaminants of the marine environment, and the deep seafloor is their ultimate sink compartment. Manipulative and field experiments provided evidence of the ingestion of MPs by deep-sea fauna, but knowledge of MPs’ fate once ingested still remains scant. We provide evidence of MP partial retention and fragmentation mediated by digestion activity of a Norwegian langoustine, a good bioindicator for MP contamination of the deep sea. We report here that MPs in the intestines were more abundant and significantly smaller (up to 1 order of magnitude in surface) than those in the stomachs. Our results show that the stomach can act as a size-bottleneck for ingested MPs, enhancing the retention of larger particles within the stomach and promoting fragmentation into smaller plastic debris, which is then released in the intestine. Our results provide evidence that the langoustine is responsible for the fragmentation of MPs already accumulated in sediments through its scavenging activity and digestion. These findings highlight the existence of a new peculiar kind of “secondary” MPs, introduced in the environment by biological activities, which could represent a significant pathway of plastic degradation in a secluded and stable environment such as the deep sea.

## Introduction

Since the 1950s, plastic production worldwide resulted in the generation of 6.3 billion metric tons (Mt) of waste, 79% of which (ca. 5 billion tons) is dispersed in the environment.^1^ It has been estimated that 5–8 Mt of plastic moves from land to oceans every year,^2^ also reaching secluded environments such as polar regions and the deep ocean floor.^3,4^ The deep ocean represents the final sink for marine litter and microplastics (MPs),^5−9^ which, due to their small size and slow weathering process,^8−12^ can be ingested by organisms^13^ even in these extreme and remote environments.^8,9,12,14^

Vagile benthic fauna is known to be particularly exposed to MP ingestion,^10,15,16^ but the fate of ingested MPs is still to be clarified, particularly in the deep sea. The size of MP particles influences their ingestion and egestion rates, and their isolation in tissues of marine organisms, by itself, does not represent a reliable proxy for particle retention or for their accumulation.

The Norwegian langoustine *Nephrops norvegicus* (L. 1758) is a benthic decapod inhabiting European temperate and cold waters. Langoustines represent a relevant fishery resource that is worth millions of euros of income for professional fishery operating in European waters: it is highly appreciated as gourmet seafood, with a market price comparable to those of other high-quality crustaceans such as lobsters, spiny lobsters, or deep-sea shrimps.^17,18^ The langoustine is a key element in muddy bottoms around European waters, especially in the Mediterranean, where its distribution is constrained to deep waters, usually below 200 m in depth. The continuous scavenging behavior on the seabed allows langoustines to interact not only with other benthic species but also with sediment-water fluxes and resuspended sediments.^19^ Because of this, *N. norvegicus* has been suggested as a reliable bioindicator of MP contamination of the deep seabed.^20,21^ The Mediterranean Sea is estimated to retain between 21 and 54% as the number of global MP particles (3.2–28.2 × 10^12^ particles), equivalent to 5–10% of the global plastic mass (4.8–30.3 × 10^3^ tons) in the oceans,^22,23^ and the resident population of *N. norvegicus* showed to diffusely ingest MPs.^21^ All the available information on the MP occurrence in this species reflects the isolation of these particles from its stomach contents or through the digestion of its entire digestive apparatus.^20,24−26^

Laboratory experiments showed that crustaceans can be able to modulate the fragmentation of MPs into smaller fragments;^27^ in addition, langoustines exposed to MPs suffer from reduced body mass and feeding and metabolic rates compared to normally fed animals.^28^ Since no evidence exists on the occurrence of these processes in the field, the aim of this work was to evaluate whether fragmentation of ingested MPs occurs also in wild organisms, thus demonstrating the existence of a new type of “secondary” plastic produced by biological activities. This would result in the re-emission of smaller and more bioavailable plastic particles that could potentially impact lower trophic levels of deep-sea food webs.

## Materials and Methods

### Specimen Collection and Treatment

Analyses were performed on stomachs and intestines of 27 langoustines collected during trawl surveys at depths between 402 and 656 m in Sardinian waters, Italy, Mediterranean Sea (Figure S1 and Table S1). Specimens were collected and transported in the laboratory for dissection to avoid the risk of contamination from sampling activities. Each stomach and intestine was dissected and stored at −20° until analysis, before which they were dried at 60 ° C for 24 h and pottered for the subsequent MP extraction.

### MP Extraction and Characterization

MP extraction was performed using the protocol cross-validated during a common initiative of two JPI Oceans Projects, namely, EPHEMARE and BASEMAN, and tested with several species, including the one targeted in the present study.^10,13,21,29,30^ Briefly, MPs are extracted from dried tissues through density separation (in NaCl saturated solution) and filtered on cellulose nitrate membranes (8 μm pore size) for the subsequent visual sorting and μ-FTIR characterization.

Before extraction and between each step of the extraction protocol, benches were cleaned with Milli-Q water. All working solutions were prefiltered through a nitrate acetate membrane with a pore size of 0.45 μm. Glass and metal materials were used and rinsed with prefiltered Milli-Q water before use. After rinsing, all containers were covered with aluminum foils, which were also kept during digestion, stirring, decantation, and filtration steps. After filtration, membranes were kept in glass Petri dishes, previously rinsed with prefiltered Milli-Q water. Cotton lab coats were used at all times, and special attention was paid to limit the wearing of synthetic clothes. NaCl saturated solution was prepared in distilled and prefiltered (0.45 μm pore size) water. Contamination controls were also included (one control for each batch of samples was treated in parallel to samples), consisting of prefiltered hypersaline solution that undertook all the drying, extraction, digestion, and sorting steps.

All particles retrieved on membranes were sorted under a stereo-microscope (64×), photographed, categorized according to shape (fragments, film, pellet/beads, and filaments) and color (white/transparent, red/orange, dark color, and others), and then measured (maximum length and surface) through the CPCe software.^31^

All extracted particles were characterized using a μFTIR microscope (Spotlight i200, PerkinElmer) coupled to a spectrometer (Spectrum Two, PerkinElmer). The measurements were made using the μATR mode. Following background scans, 32 scans were performed for each particle, with a resolution of 4 cm^–1^. Spectrum 10 software was used for the output spectra, and the identification of polymers was performed by comparison with libraries of standard spectra. Polymers matching for more than 70% with the reference spectra were validated, while polymers with a match between 60 and 70% underwent a critical interpretation of the spectra.^10^

Despite the above-described precautions, it was not possible to fully avoid airborne contamination, and some textile fibers were found in the control membranes: the μFTIR characterization revealed these fibers to be non-synthetic and almost constituted of cotton and wool. For this reason, they were not included in the presented results.

### Statistical Analyses

We tested our hypothesis through PERMutational ANalysis Of VAriance (hereafter PERMANOVA)^32^ using the factor “stomach/intestine” as the unique source of variation. In detail, we tested for significant differences in (i) the number of MPs in stomach^–1^ or intestine^–1^, (ii) particle size (as both maximum length and total surface), and (iii) polymer composition between the stomach and intestine. The analyses were carried out on Euclidean distance-based resemblance matrixes of untransformed data.

## Results and Discussion

All 27 *N. norvegicus* specimens had ingested MPs (100% occurrence), with 18 stomachs out of 27 (ca. 70%) and 24 intestines out of 27 (89%) containing at least one particle. In detail, nine individuals had stomachs free of MPs but contained MPs in their intestines, and only three had MPs in the stomach but not in the intestine. Since the majority of previous studies have searched for MPs only in stomachs, our results indicate that the occurrence of MPs in this species could have been underestimated and that the contamination of *N. norvegicus* could be more severe than previously thought, at least for the Mediterranean area.

**Figure 1:**
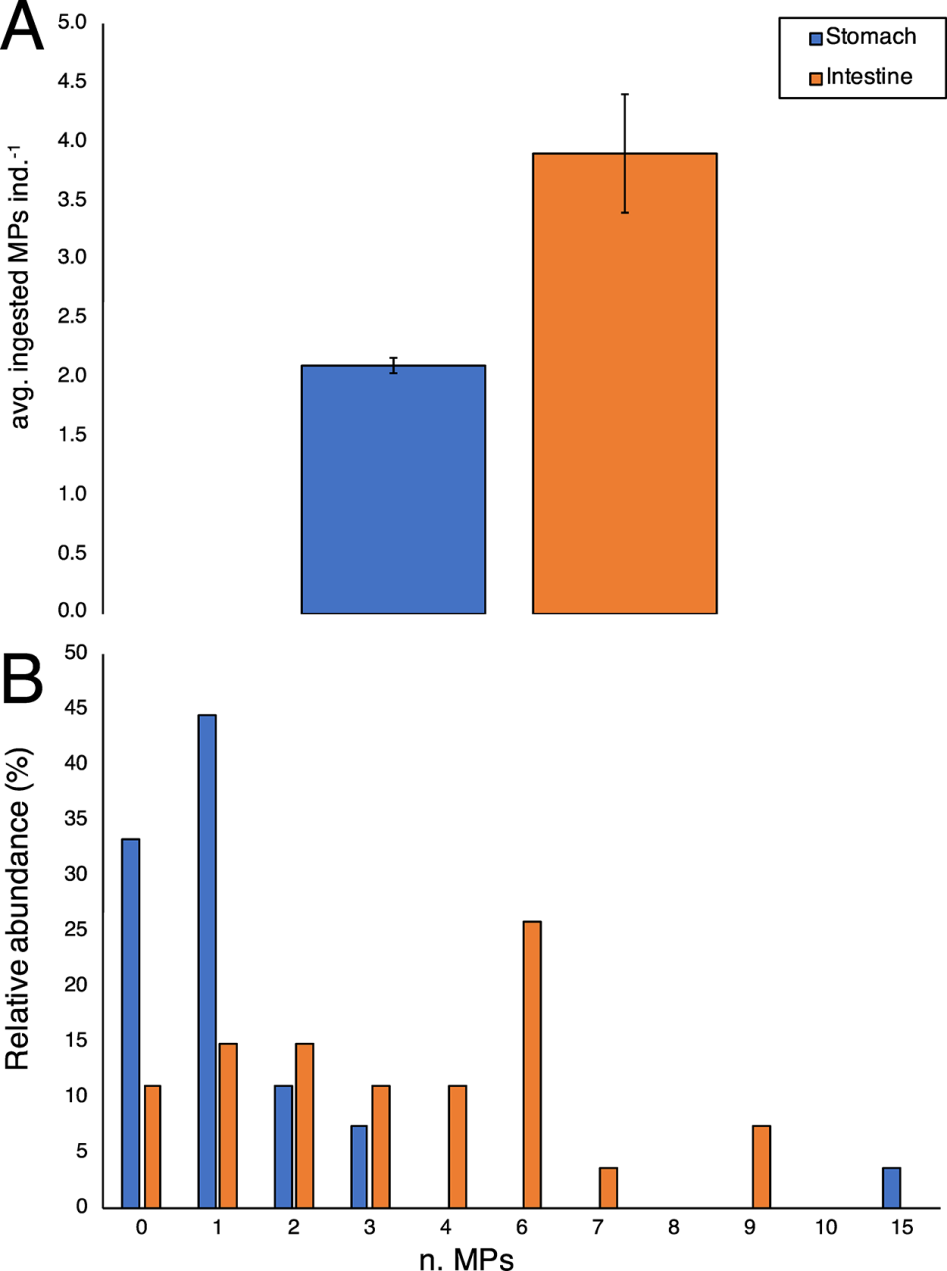
(A) Average number of MPs extracted from the two organs. (B) Relative abundance of MPs isolated from *N. norvegicus* stomachs and intestines.

A total of 730 MP-like particles were extracted from the stomachs and intestines of *N. norvegicus*, 19% of which (37 in stomachs and 94 in intestines) were actually made of plastic. Considering only the positive specimens, the average numbers of MPs were 2.1 ± 0.6 MPs and 3.9 ± 0.5 MPs in stomachs and intestines, respectively (Figure 1A). In detail, the number of MPs in stomachs ranged from 0 to a maximum of 13 particles per sample, whereas in intestines, MP abundance ranged from 0 to 9 particles per sample (Figure 1B). In general, 85% of analyzed specimens had more MPs in the intestines than in the stomachs (Figure S2), indicating that a large proportion of ingested MPs leaves the stomach and enters the intestine, without any further retention in the first part of the digestive apparatus (Figure S3). With respect to the color of MPs, in both compartments, white/transparent items were the most common (with 81 and 68%, respectively) followed by dark (13 and 12%) and red (5 and 15%) (Figure S4). Plastic fragments were the most abundant typology of MPs (62 and 72% in stomachs and intestines, respectively) followed by films (38 and 12% in stomachs and intestines, respectively).

**Figure 2:**
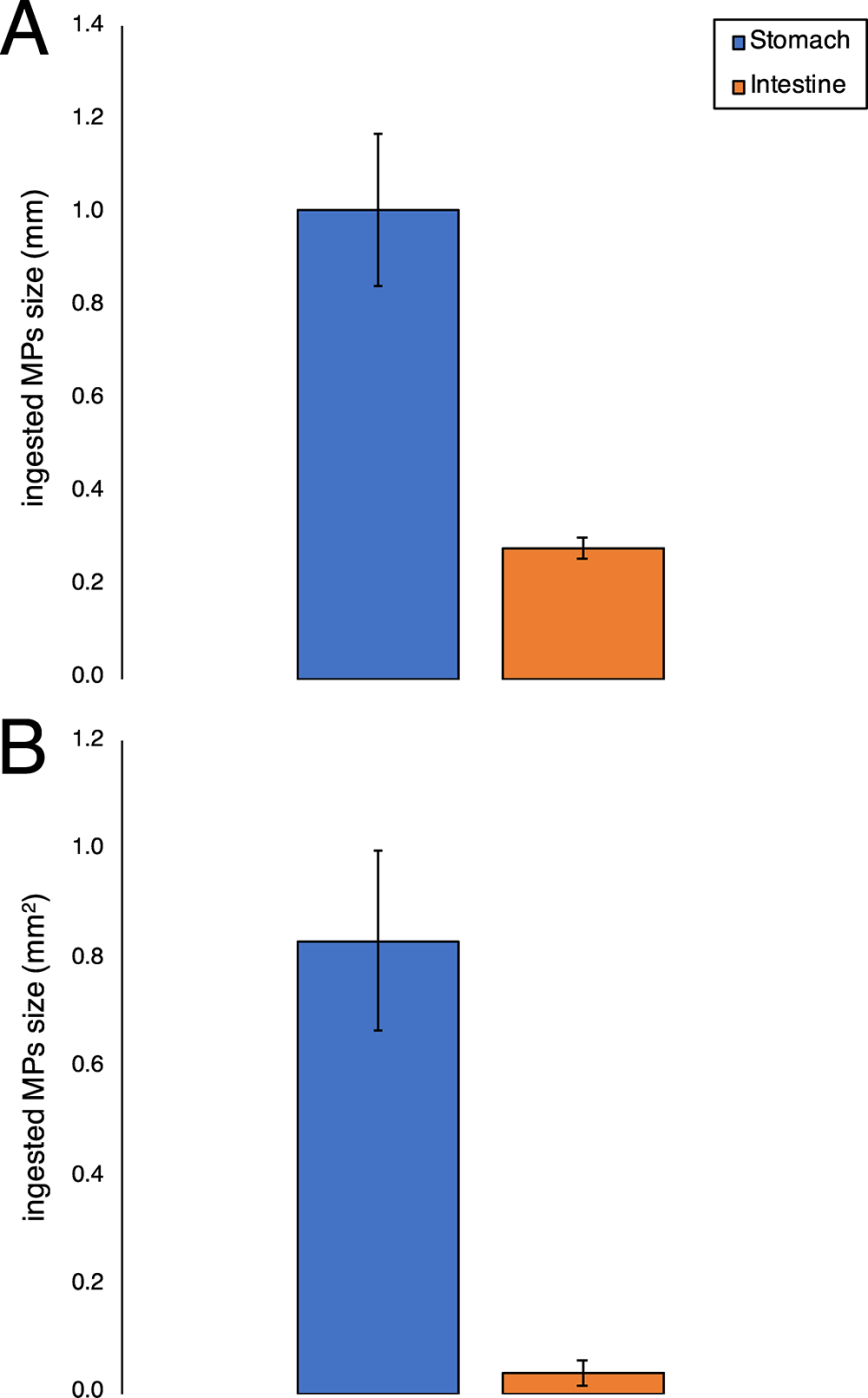
Histograms showing different average sizes (A, in mm) and surfaces (B, in mm*^2^*) of particles isolated from stomachs and intestines of *N. norvegicus*.

**Figure 3:**
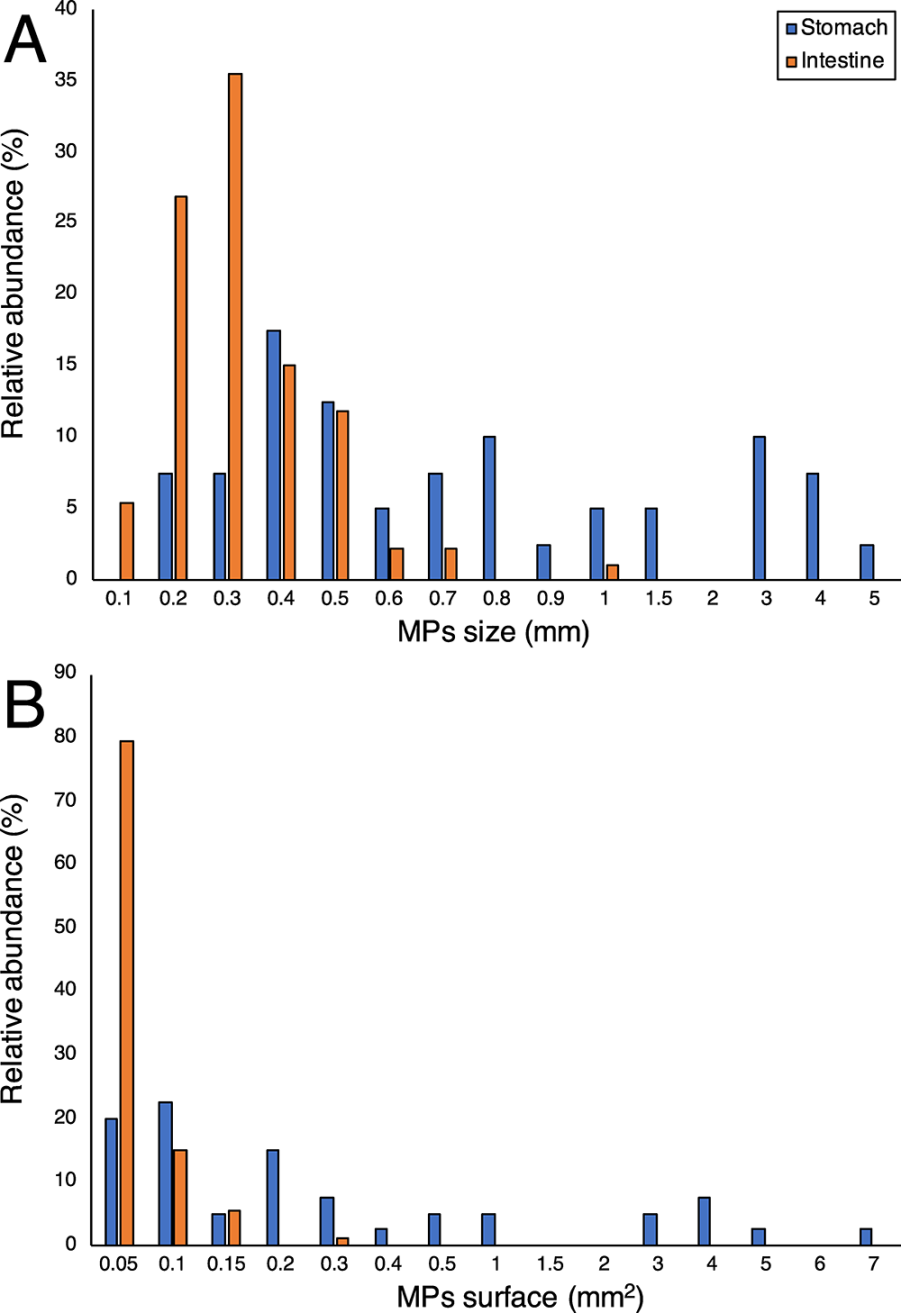
Percentage distributions of (A) MP size (maximum length, in mm) and (B) surface (in mm^2^) isolated from stomachs and intestines in *N. norvegicus*.

MPs isolated from intestines exhibited a maximum length (0.23 ± 0.16 mm) and surface (0.04 ± 0.005 mm^2^) significantly smaller than those found in stomachs (1 ± 0.16; 0.83 ± 0.25 mm^2^) (Table 1, Figure 2). The differences in maximum length (400% smaller in the intestine) and surface (an order of magnitude smaller in intestines) were consistently observed in all specimens analyzed (Figure S5). Further, the MP size frequency distributions in the stomach and intestine (Figure 3) indicated that smaller particles with a maximum length of <0.5 mm represented ca. 94% of MPs isolated from intestines and only 45% of those from stomachs (Figure S6A), whereas MPs with an average surface of <0.1 mm^2^ represented ca. 94 and 42% of the MPs in the intestines and stomachs, respectively (Figure S6B). The predominance of larger fragments and films confined within the stomach could be explained by the peculiar conformation of the digestive system of these crustaceans:^25^ indeed, ingested food particles are broken up by the gastric mill, a complex of small calcified plates moving against each other for grinding. Triturated particles then pass through a complicated filter apparatus of setae, which allows only the smaller particles to pass through the mid-gut and consequently into the hind-gut^19,^^33^(see Figure S3). In this respect, the filter apparatus represents a sort of bottleneck impeding the transit of larger particles toward the intestine until they are fragmented enough to pass the cardio-pyloric valve (Figure S3).

**Table 1:** Results from the PERMANOVA Testing for Differences in the Number, Polymeric Composition of Ingested MPs, and Size of MPs between the Stomachs and Intestines of *N. norvegicus*

MP abundance (no. of particles stomach^–1^ or TD^–1^)				
source	df	MS	pseudo-*F*	*P* (MC)
stomach/TD	1	34.9	5.49	0.025
res	41	6.33		
total	42			

MP polymer composition				
source	df	MS	pseudo-*F*	*P* (MC)
stomach/TD	1	16.09	2.84	0.031
res	41	5.65		
total	42			

MP size (mm)				
source	df	MS	pseudo-*F*	*P* (MC)
stomach/TD	1	14.182	41.08	0.001
res	132	0.345		
total	133			

MP surface (mm^2^)				
source	df	MS	pseudo-*F*	*P* (MC)
stomach/TD	1	17.77	23.711	0.001
res	132	0.749		
total	133			

**Figure 4:**
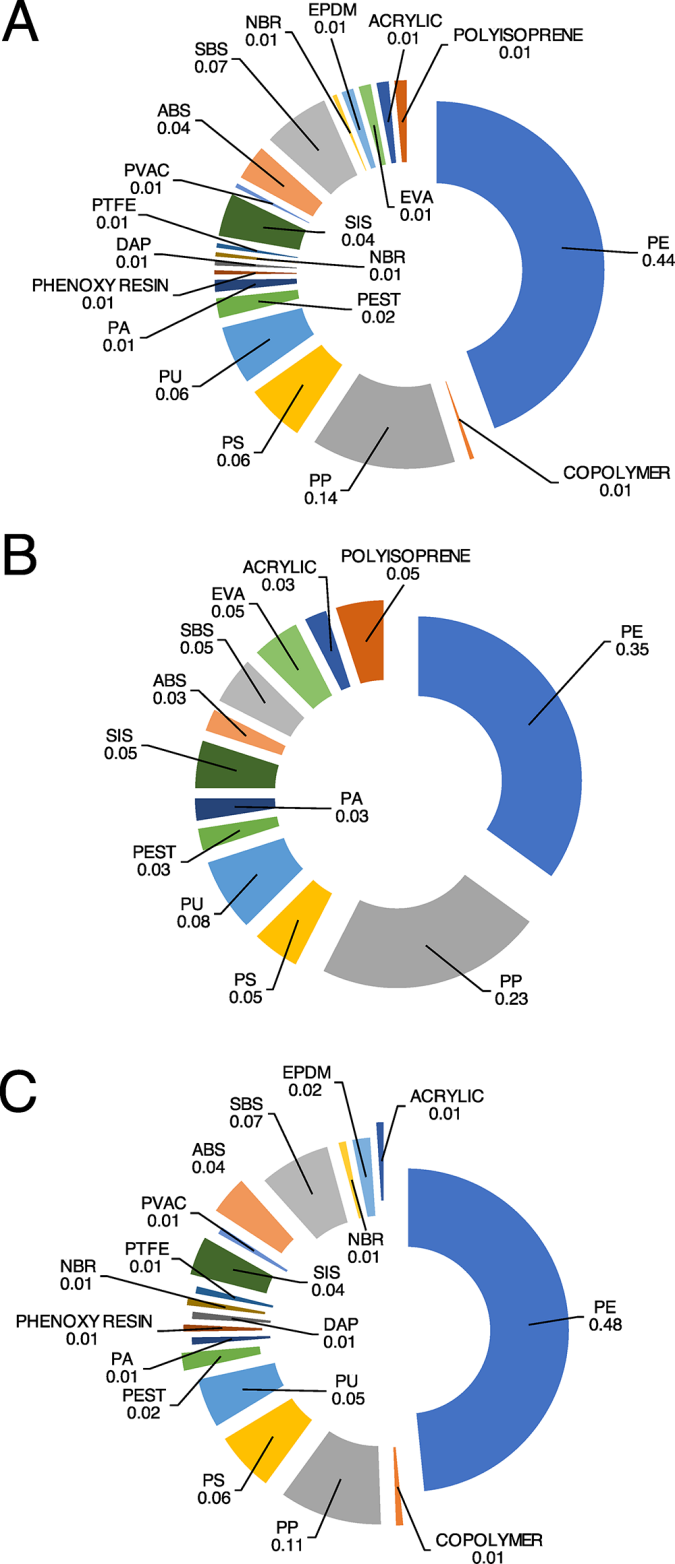
Polymeric composition (%) of MPs retrieved from *N. norvegicus*: (A) overall composition, (B) MP composition in stomachs, and (C) MP composition in intestines.

Overall, polyethylene, polypropylene, and polystyrene accounted for ca. 65% of the retrieved MPs (Figure 4A). Considering the polymeric composition, shape, and color of extracted particles, we can suppose that the majority are derived from packaging materials. Previous surveys reported a particularly severe presence of single-use plastic materials in the investigated depth range for the whole Mediterranean area, possibly due to the close proximity of submarine canyons that can act as both traps and conduits of marine debris toward the deep sea.^5,7,21^ MPs in stomachs and intestines showed significant differences in polymer composition (Table 1). MPs isolated from stomachs contained 12 typologies of polymers, while 18 polymers were detected in intestines (Figure 4B,C), which contained also noncommon polymers, such as acrylonitrile butadiene styrene (ABS), styrene-isoprene-styrene (SIS), acrylonitrile butadiene rubber (NBR), polyester (PEST), acrylic, polyvinyl alcohol (PVA), polytetrafluoroethylene (PTFE), phenoxy resin, and diallyl phthalate (DAP). The exclusive presence of these polymers in MPs retrieved from the intestines is likely associated with smaller particles that pass unscathed through the stomach’s filter apparatus. Overall, the predominance of transparent particles composed of polymers belonging to polyolefin classes PE and PP allows us to hypothesize that disposable materials, such as bags and containers, may be the dominant sources of MPs.

The capability of crustaceans to triturate and reduce the size of ingested plastic fragments has been previously documented through the use of fluorescent particles in laboratory experiments.^27^ Histological analyses documented the co-occurrence of both large and smaller particles (i.e., both entire and fragmented ones) within the digestive apparatus and in feces. Despite the lack of information about the time that langoustines might need to triturate larger particles, the analysis of langoustine specimens (*n* = 7) containing particles with the same shape, polymeric composition, and color (all transparent PE and PP films) in both stomach and intestine revealed that the size of those particles decreased consistently from the stomach to the intestine (Figure S7).

The results presented in this study are the first documentation that the peculiar digestive behavior of *N. norvegicus* can be responsible for the fragmentation and redistribution of smaller secondary MPs in the environment, thus modulating and extending their environmental path. Some models have conservatively estimated that over 5 trillion of plastic items float over the oceans’ surface, which however accounts for only 1% of ocean plastic load, thus indicating that the deep seafloor is the major plastic debris repository, where they persist and accumulate.^5,7,34,35^ At this time, it is generally accepted that plastic reaching the seafloor might undergo slow environmental degradation, be permanently buried and accumulated in deep-sea sediments,^5,36^ or be accidentally ingested by deep-sea fauna and eventually transferred to higher trophic levels.^12,34^ Our results document the existence of a further possibility: the biologically mediated redistribution of progressively smaller MPs in the environment, suggesting that this species could potentially enhance both plastic environmental degradation^37^ and its availability to smaller fauna. Nano- and microplastics can thus become more bioavailable through accidental ingestion since the particles are of roughly the same size (or even smaller) as sediment grains^38^ which implies a potential impact on trophic energy transfer and/or trophic interactions.

Our results empirically demonstrate for the first time the occurrence of this phenomenon in the field*,* possibly representing a significant and underestimated pathway for the degradation of plastic debris in the marine environment. In addition, our findings may pose the question on what actually could be the effective proportion of “biologically mediated” secondary MPs present in deep-sea environments, at least in those where scavenging crustaceans are present.

Concerning larger particles retained in the digestive apparatus, our results do not allow assessment of how long it might take for particles to be further fragmented and excreted; however, manipulative laboratory experiments already showed that long-term plastic-fed langoustines suffered from higher mortality, false satiation, and disrupted assimilation of food, ultimately influencing biological traits such as growth and reproduction.^28^ Since these authors commented their results as conceivably representative of those experienced by animals dwelling in highly contaminated areas, our findings, showing even higher contamination records, pose the question as to whether plastic contamination of wild crustaceans may have similar effects on their natural feeding and reproduction capability, with consequences on the overall health of commercial stocks. In addition, the passage of small MPs through the intestine could induce more ecotoxicological effects than larger particles.^39,40^ Despite not being detected by our extraction protocol, it is worth pointing out that nanoparticles, which are likely to be produced through the process here described, can potentially pass intestinal barriers of animals (including humans). Marine organisms could thus be subjected to a chronic presence of such particles, increasing the intensity of sublethal effects usually observed after an MP exposure such as the onset of neurotoxic, genotoxic, and oxidative damage.^41−44^

In conclusion, our study documented the occurrence of biological fragmentation of microplastics by deep-sea crustaceans, highlighting the need for further research to characterize the role of benthic fauna in modulating weathering of MPs, the proportion of this phenomenon in the marine environment, and the potential interaction between biologically fragmented MPs and trophic webs.

## Supporting Information Available

Details and map of the sampling area; a scheme showing the path of microplastics in the digestive apparatus of *N. norvegicus*; histograms showing the different sizes of microplastics isolated from stomachs and intestines (PDF)
