# Supplementary material for: Benthic Crustacean Digestion Can Modulate the Environmental Fate of Microplastics in the Deep Sea
Source: Environ Sci Technol. 2020 Mar 19;54(8):4886–92. doi: 10.1021/acs.est.9b07705 (PMC7997365; doi:10.1021/acs.est.9b07705)
Supplement: Supplementary file 1 — es9b07705_si_001.pdf [file es9b07705_si_001.pdf]

SUPPORTING INFORMATION FOR:

Benthic crustacean digestion can modulate environmental fate of microplastics in the deep sea

Alessandro Cau*[1,2]✉, Carlo Giacomo Avio*[3], Claudia Dessì[1], Davide Moccia[1], Antonio Pusceddu[1,2], Francesco Regoli[3,4], Rita Cannas[1,2] & Maria Cristina Follesa[1,2]

[1]Università degli Studi di Cagliari, Dipartimento di Scienze della Vita e dell'Ambiente, Via Tommaso Fiorelli 1, 09126 Cagliari, Italy

[2] Consorzio Interuniversitario per le Scienze del Mare, CoNISMa, ULR Cagliari, Cagliari, Italy

[3]Università Politecnica delle Marche, Dipartimento di Scienze della Vita e dell'Ambiente, Via Brecce Bianche, 60131, Ancona, Italy

[4] Consorzio Interuniversitario per le Scienze del Mare, CoNISMa, ULR Ancona, Ancona, Italy

*= equally contributing authors.

✉**Corresponding Authors:**

Dr. Alessandro Cau

Telephone: +39 070 675 6626

E-mail: alessandrocau@unica.it;

Number of pages: 9

Number of tables: 1

Number of figures: 7

SUPPORTING INFORMATION FOR:

Table 1. Number of individuals, geographical coordinates and average depth of trawls conducted in the 3 sampling sites.

Site	n. of individuals	Latitudine (N)	Longitude (E)	Average depth (m)
1	10	40° 30' 40.8"	7° 54' 15.6"	402
2	8	40° 36' 07.8"	7° 49' 58.2"	656
3	9	41° 10' 42.0"	8° 47' 37.2"	415

SUPPORTING INFORMATION FOR:

st[illegible]

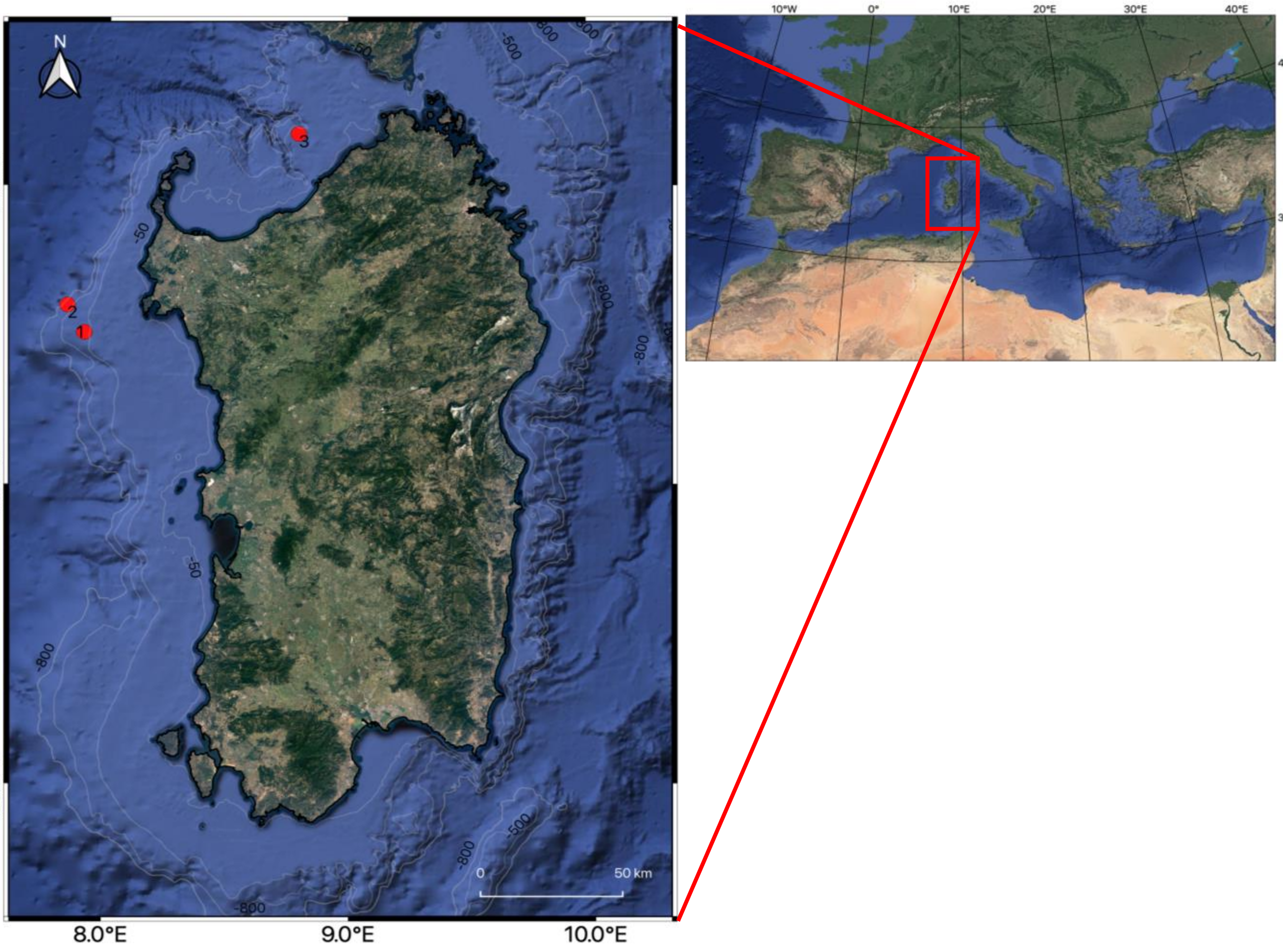

Figure S1. Map of the investigated area.

SUPPORTING INFORMATION FOR:

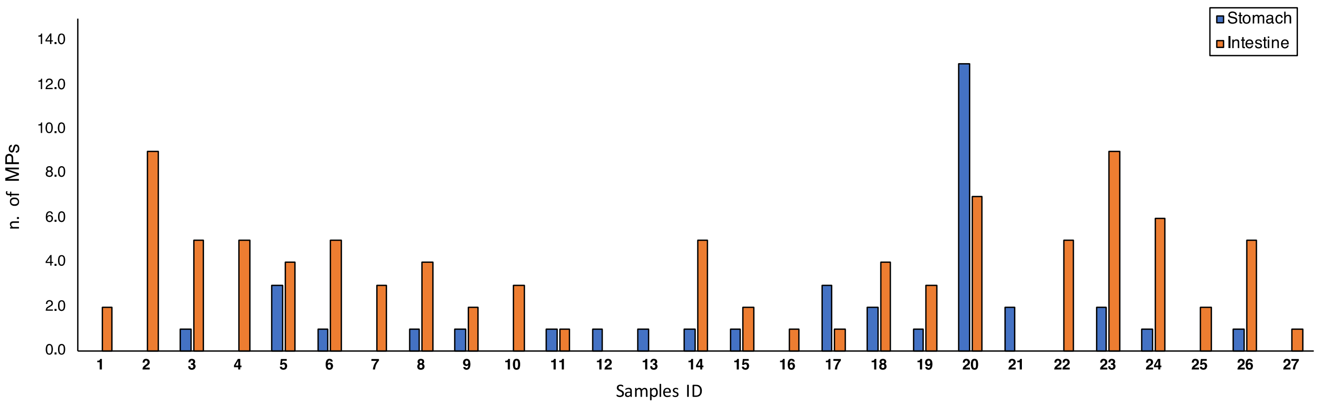

Figure S2. Histogram showing the number of MPs isolated from stomach and intestine in each sample considered in the present

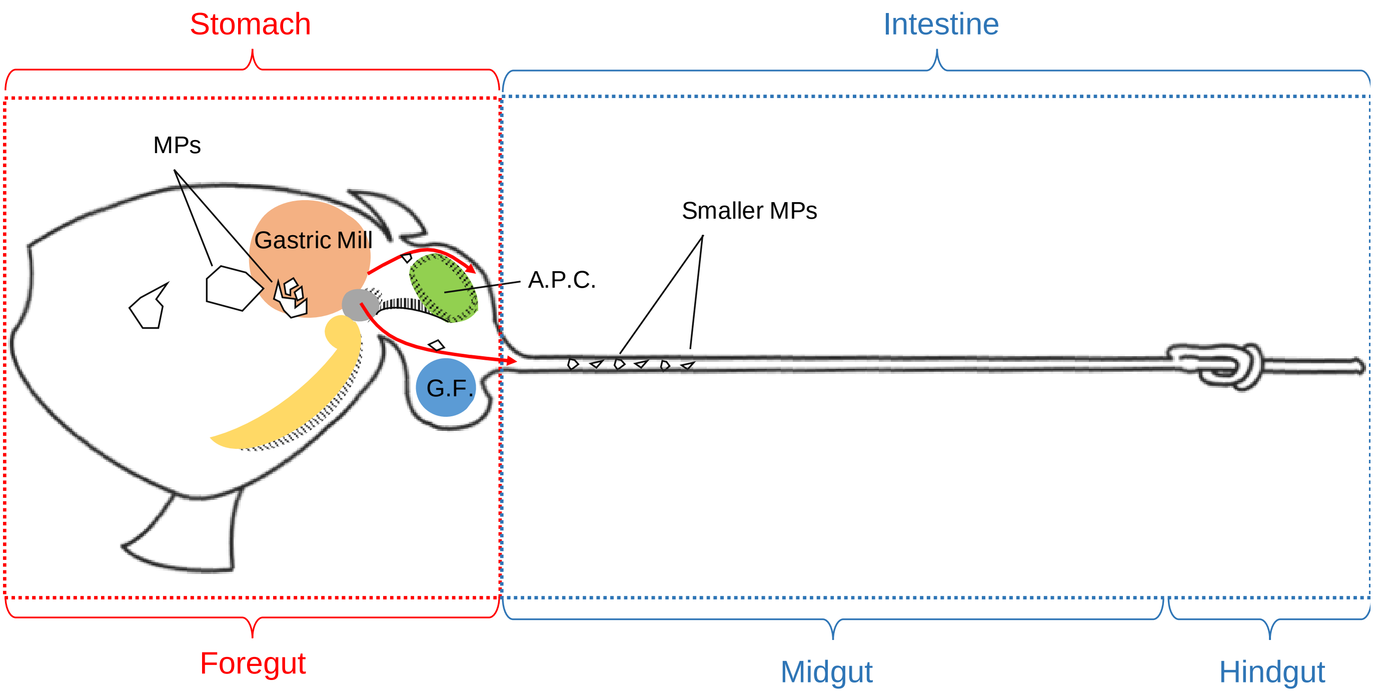

Figure S3. The path of MPs in the digestive tract of *Nephrops norvegicus*: i) once ingested, MPs are fragmented into the gastric mill; ii) reduced particles move through the filter apparatus, above the anterior pyloric chamber (A.P.C.) and the gland filter (G.F.), contained into the foregut; iii) the fraction of smaller MPs goes to the midgut and consequently to the hindgut (intestine).

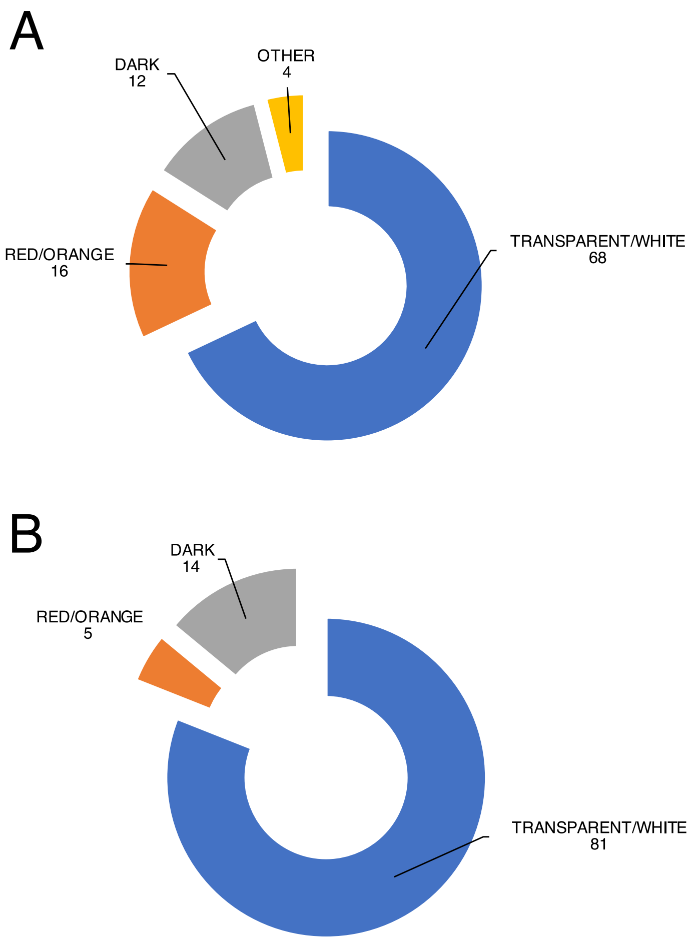

Figure S4. Color composition (%) of MPs retrieved from *N. norvegicus* stomachs (A) and intestines (B).

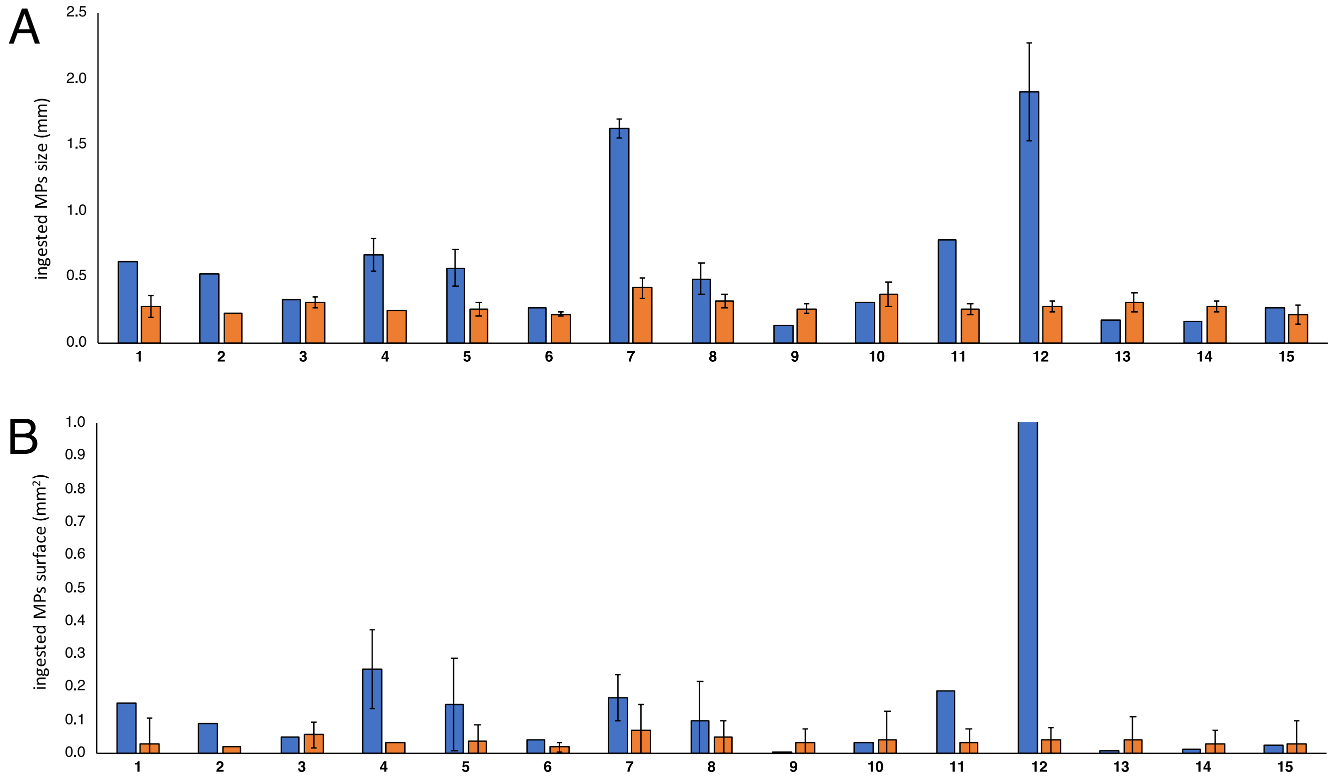

Figure S5. Histogram showing the different size and surface of particles from individuals showing the presence of MPs in both stomachs and relative intestines.

SUPPORTING INFORMATION FOR:

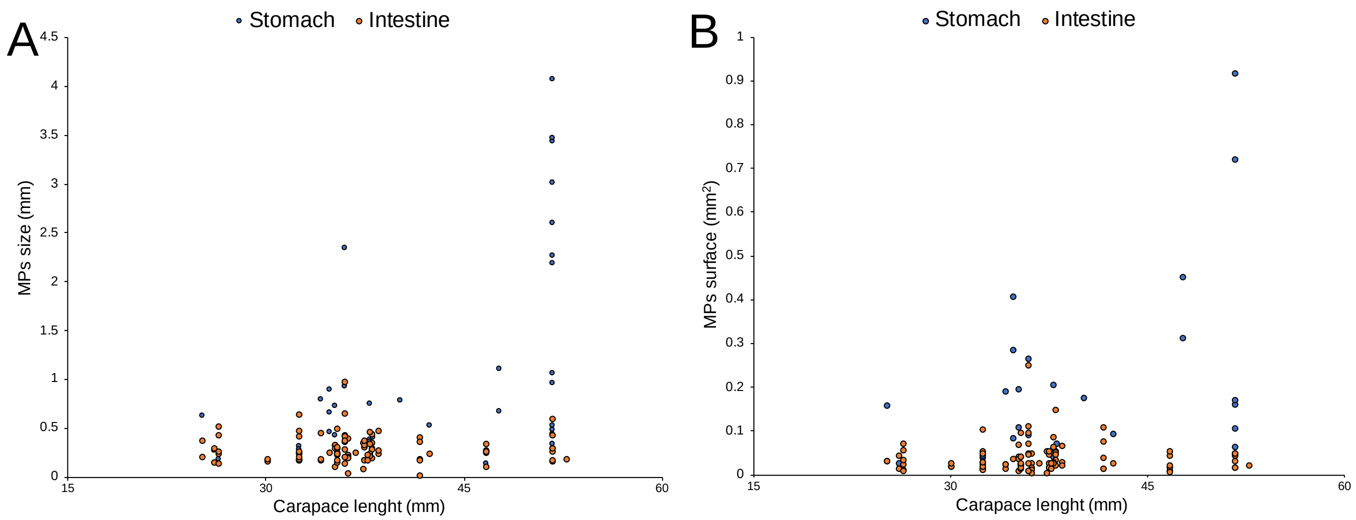

Figure S6. Histogram showing the size and surface of all particles isolated from stomachs and relative intestines of *N. norvegicus*. The orange and blue shadows represent the size and surface range comprising >90% of measured particles, for both stomach and intestine. Measures are correlated with Carapace Length of individuals in abscissa, showing the absence of correlation between the size of individuals and the size or surface of ingested MPs in intestines.

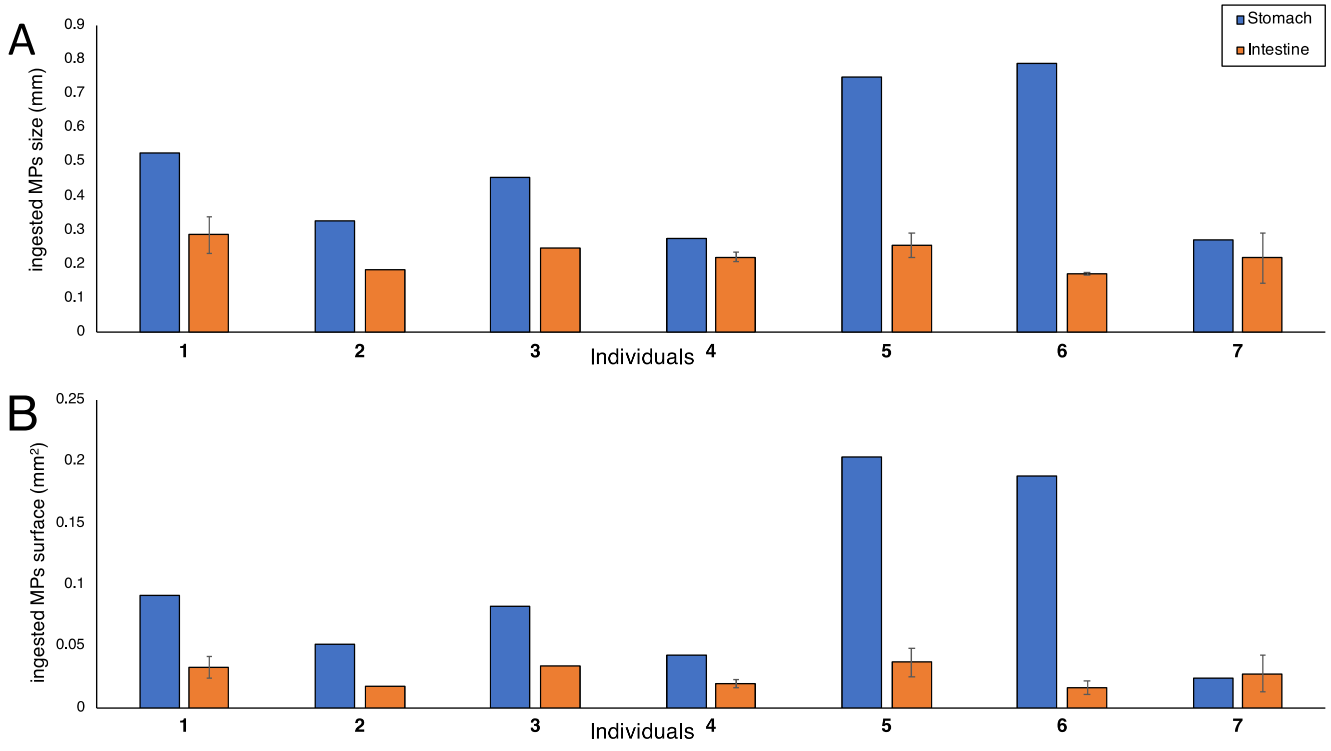

Figure S7. Histogram showing the different size and surface of particles of the same polymeric nature isolated from stomachs and relative intestines. MPs from stomach and relative intestine were classified of same polymeric nature only if the polymer type, shape and colour was the same; it was possible to perform this comparison in 7 individuals out of the total. Error bars are reported when more than one particle was isolated and measured.
